# Synthesis, Characterization and In Vitro Cytotoxic Assay of La(III) Complex with Chlorambucil

**DOI:** 10.3390/molecules31203584

**Published:** 2026-10-09

**Authors:** Fernanda Timoteo, Kristiane Fanti Del Pino, Danielle Bogo, Vasilii Khripun, Franciele Garcia Baveloni, Sabrina Epifânio Soares de Souza, Brunna Mercedes Barboza Borges, Guilherme Pereira Biazoto, Diego Boldo, Adriana Pereira Duarte, Adriana da Silva Posso, Marco Antonio Utrera Martines

**Affiliations:** 1 Chemistry Institute, Federal University of Mato Grosso do Sul, Campo Grande 79079-900, MS, Brazil; fernanda.timoteo@ufms.br (F.T.); kristianefdp@gmail.com (K.F.D.P.); vasilli.khripun@ufms.br (V.K.); franciele.baveloni@ufms.br (F.G.B.); sabrinaepsouza@gmail.com (S.E.S.d.S.); brunna.borges@ufms.br (B.M.B.B.); guilherme.biazoto@ufms.br (G.P.B.); diego.boldo18@gmail.com (D.B.); adriana.duarte@ufms.br (A.P.D.); adriana.posso@ufms.br (A.d.S.P.); 2 Faculty of Pharmacy, Food and Nutrition, Federal University of Mato Grosso do Sul, Campo Grande 79079-900, MS, Brazil; danielle.bogo@ufms.br

**Keywords:** lanthanum chlorambucil complex, in vitro cytotoxic assay, anticancer activity assays, DFT calculations

## Abstract

This work studies the synthesis of complexes using chlorambucil (La-CLA) as a ligand and lanthanum as a metallic center, in order to enhance their biological activities. The compounds produced were characterized by infrared spectroscopy and 13C and 1H nuclear magnetic resonance; the results obtained indicate that coordination bonds formed between the ligands and that La3+ was present in a bidentate way through carboxylic groups in the complexes. Elemental and thermogravimetric analyses indicate that the stoichiometry obtained for chlorambucil complexes was a 2:1 ligand:metal ratio. The anticancer activity assays indicated that the La-CLA complex showed better activity than the ligands against the cell lines K562 (Chronic Myeloid Leukemia), HT-29 (Colorectal Cancer), Caco 2 (Human Colorectal Adenocarcinoma) and B16-F10 (Murine Melanoma), with GI50 values in the range of 0.31–31.86 μM. The La-CLA complex showed a selectivity index (SI) equal to 15.64 for the K562 cell line with respect to normal J774 cells (murine macrophages).

## 1. Introduction

Currently, cancer is defined as a disease characterized by the uncontrolled proliferation of transformed cells subjected to evolution through natural selection. This process involves the accumulation of genetic and phenotypic alterations that promote cell survival, adaptation, and tumor progression, ultimately leading to tumor development and, in advanced stages, metastasis [1]. According to the World Health Organization (WHO), the global incidence of cancer is projected to reach 30 million new cases by 2040, representing a 56.2% increase compared with the 19.2 million cases reported in 2020. Furthermore, a WHO survey conducted in 2024 revealed that cancer was the first or second leading cause of death in 134 countries and ranked third or fourth in an additional 45 countries [2].

In this context, the synthesis of new compounds for the treatment of these diseases has become an important focus of research. Among the strategies employed in the search for novel pharmacological prototypes, structural optimization of molecules with well-established biological activity has been widely explored in order to enhance therapeutic efficacy while reducing adverse effects [3].

Since the development of a new drug from discovery to commercialization is a lengthy, challenging, and costly process that may exceed ten years [4], chemical modification of compounds already used as active pharmaceutical ingredients or possessing well-characterized biological activity has emerged as an effective approach to accelerate drug development [5]. In this regard, although only a limited number of metal complexes are currently used as therapeutic agents, this field presents considerable potential, as several studies have demonstrated enhanced biological activity following complexation with lanthanide ions [6,7,8,9], with some compounds exhibiting greater activity than the reference drug cisplatin [10]. To date, only a limited number of lanthanide-based compounds have achieved clinical application. Among them, Lutathera ([177Lu]Lu-DOTA-TATE), a lutetium-based radiopharmaceutical, was approved by the FDA in 2018 for peptide receptor radionuclide therapy (PRRT) [11]. Another lanthanide-containing radiopharmaceutical currently used in clinical practice is samarium-153 lexidronam, which is employed for the palliative treatment of bone metastases [12].

Considering the therapeutic potential of metal-based compounds, the development of new coordination complexes using biologically active ligands has emerged as a promising strategy for anticancer therapy. In this context, chlorambucil (CLA), a clinically established antineoplastic agent, was selected as the ligand due to its well-characterized biological activities. Previous studies have demonstrated that metal coordination may significantly enhance the antitumor activity of these compounds. Qin et al. [13], for example, reported platinum–chlorambucil complexes with greater cytotoxic activity than their precursors, including activity against cisplatin-resistant gastric carcinoma cells. In parallel, lanthanide ions have attracted considerable attention in medicinal chemistry due to their physicochemical properties and biological relevance. Owing to their ionic radius similarity to Ca^2+^, Ln^3+^ ions can interact with calcium-binding sites and interfere with cellular signaling pathways [14,15,16,17,18], contributing to anticancer effects such as apoptosis induction and tumor growth inhibition [19,20]. Moreover, several lanthanum-based complexes have demonstrated expressive cytotoxic activity against different cancer cell lines, including cisplatin-resistant tumors, while maintaining low toxicity toward normal cells [21,22]. Therefore, the synthesis of lanthanum complexes containing CLA ligands represents a promising approach for the development of new anticancer agents with enhanced biological activity and potential to overcome drug resistance mechanisms.

## 2. Results and Discussion

### 2.1. Characterization of La(CLA)_2_(H_2_O)Cl Complex

As suitable single crystals of La(CLA)_2_(H_2_O)Cl for X-ray diffraction analysis could not be obtained, the complex was characterized using a combination of spectroscopic and thermal techniques, including Fourier-transform infrared spectroscopy (FTIR), ^1^H and ^13^C nuclear magnetic resonance (NMR) spectroscopy, and thermogravimetric analysis (TGA). These complementary techniques were employed to elucidate the structural features of the synthesized complex and to investigate the coordination mode of CLA toward the lanthanum (III) center.

**Figure 1:**
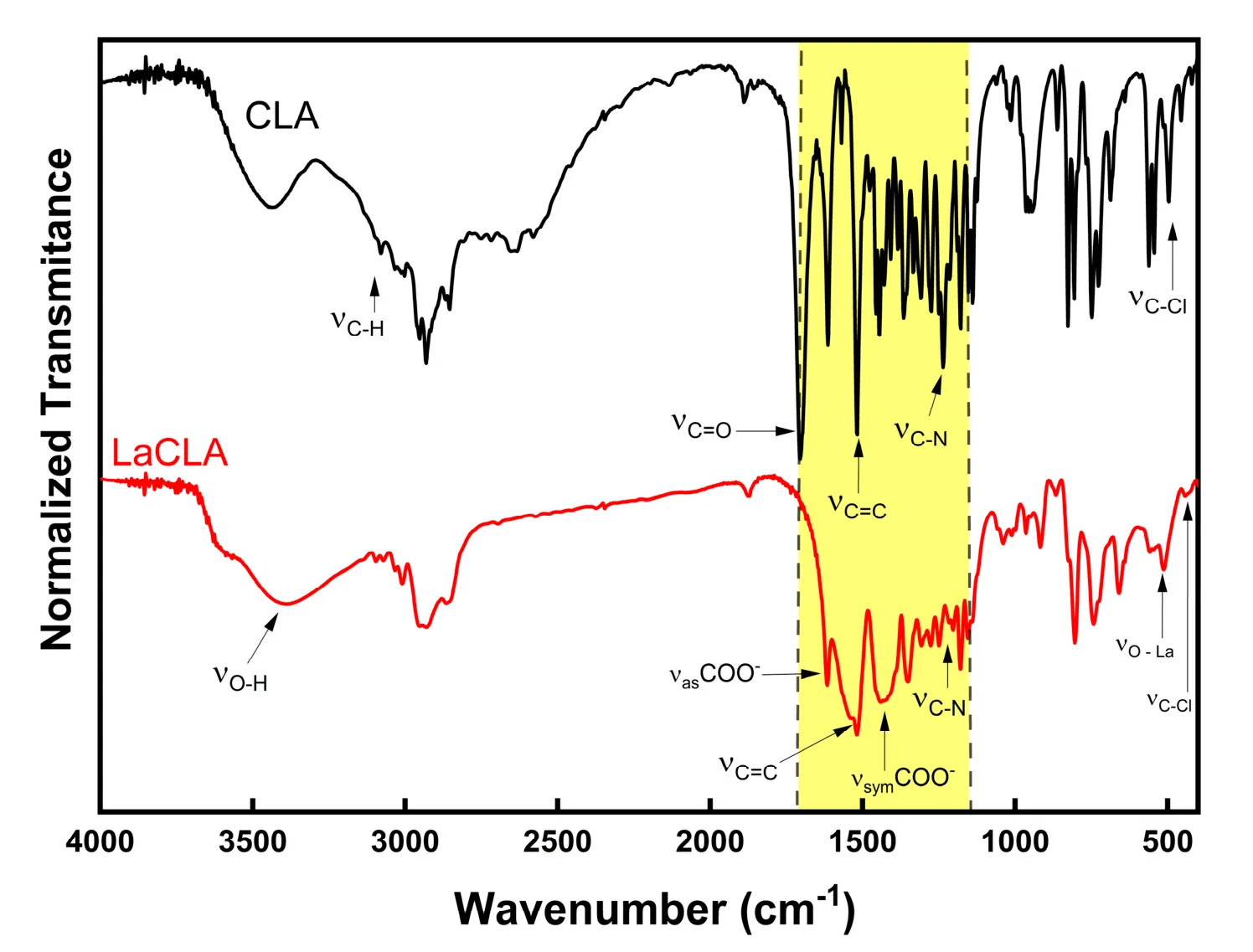
Fourier-transform infrared (FTIR) spectra of chlorambucil (CLA) and lanthanum–chlorambucil complex (La-CLA), recorded in the range of 4000–500 cm^−1^.

Infrared spectroscopy was initially used to identify the ligand coordination site. During the synthesis, triethanolamine (TEOA) was added as a base to promote deprotonation of the chlorambucil carboxylic group, thereby facilitating its coordination to the metal ion. However, TEOA contains potential donor atoms, namely the oxygen atoms of its hydroxyl (–OH) groups and the nitrogen atom, which may also interact with La^3+^ ions [23,24,25,26,27]. To assess the possible involvement of TEOA in the coordination sphere of the metal center, its infrared spectrum was included in the comparative analysis. The obtained vibrational spectra are presented in Figure 1, and the assignments of the main absorption bands are summarized in Table S1.

Analyzing the chlorambucil spectrum in Figure 1, absorption at 3442 cm^−1^ refers to the stretching of the –OH bond of the carboxylic group. The broad band is an indication that the ligand is in a dimerized form or indicates the presence of intramolecular hydrogen bonding [28]. Still associated with the carboxylic group, the intense absorption at 1705 cm^−1^ refers to the stretching of the –C=O bond. The stretching of the C-H bonds (sp2) appears at 3080 cm^−1^, which, accompanied by the symmetric (ν_s_) and asymmetric (ν_ass_) stretching of the C=C bonds observed at 1456 cm^−1^ and 1612 cm^−1^, respectively, characterize the aromatic ring in the structure [28,29].

Absorptions below 3000 cm^−1^ are stretching of the –C-H bonds of sp3 carbon, referring to the –CH_2_ groups (from 2931 cm^−1^ up to 2854 cm^−1^). The stretching of the –C-N and –C-Cl bonds can be observed at 1252 cm^−1^ and 561 cm^−1^, respectively [28,29,30].

In the spectrum of the La(CLA)_2_(H_2_O)Cl complex, a broadening of the bands and a certain similarity in the absorption profile when compared to the CLA spectrum can be noted. The band at 3423 cm^−1^ corresponding to the stretching of the –OH bond continues to be observed in the spectrum after the formation of the complex. However, in this case, we can attribute it to the coordination water present in the synthesized material. The absorptions related to the aromatic ring as well as the groups –CH_2_, –C-N and –C-Cl also appear in the spectrum of the complex and without large shifts in the wavenumber. Thus, the chemical structure of chlorambucil remains the same (see Table S1).

The most relevant change that was observed was the shift of the absorption corresponding to the –C=O group to a lower wavenumber, considering that in the CLA spectrum it was observed at 1705 cm^−1^ and in the La(CLA)_2_(H_2_O)Cl complex at 1539 cm^−1^. This shift is in accordance with the literature for complexes coordinated to Ln^3+^ via a carboxylic group [8,31]. The bands located around 1600 cm^−1^ refer to the asymmetric stretching and symmetric elongation of the carboxyl group υ(COO^−^), which is present in both the ligand and the complexes, but with slight shifts to lower wavenumbers, also indicating its participation in the coordination process [32,33]. Moreover, according to the criteria established by Deacon and Phillips [34], the difference between the asymmetric and symmetric stretching frequencies (Δν = ν asym − ν sym) of 140 cm^−1^ provides insight into the coordination mode. In the present case, the observed Δν is below 200 cm^−1^, which is consistent with a bidentate coordination of the carboxylate group. Also, the band located at 525 cm^−1^ is attributed to the La–O stretching vibration [35].

It is worth mentioning that the absorption at 1655 cm^−1^ was attributed by Haron and collaborators as an overtone of the –OH band in the TEOA spectrum; the author also reports the presence of this band in the spectrum of the complex formed between TEOA and Ln^3+^ [36]. The absence of this band in the IR spectrum of the La(CLA)_2_(H_2_O)Cl complex means that TEOA did not coordinate to the metal ion and did not enter the inner sphere of the complex.

Figure S1 illustrates a comparison between the ^1^H NMR spectra for the CLA ligand and the La(CLA)_2_(H_2_O)Cl complex with atom numeration, and the assignment of the peaks is summarized in Table S2. The spectrum of the complex exhibits all the same signals present in the spectrum of the ligand. The aromatic ring signals (H-2’ and H-3’ according to Figure S1 and Table S2) are located at 6.45 and 6.49 ppm, substituent signals of the amino group (H1a’ and H2a’) are located at 3.47 and 3.55 ppm, and the backbone resonances (H-2, H-3 and H-4) appear at 2.34, 1.74, and 2.28 ppm, respectively. The largest signal shift in the spectrum of the complex relative to the spectrum of the free ligand is observed for H-2, as this specific proton is closest to the coordinated metal center. The shifts of the remaining signals are less significant (Table S2).

Undoubtedly, the signals related to the hydrogens of the ligand also appear in the spectrum of the complex, but with a change in the profile characterized by a broadening of the signals, which precludes the analysis of the signals’ fine structure, while in the ^1^H NMR spectrum of the ligand the signals are well defined [37].

The phenomenon of signal broadening in metal complexes is generally reported in the literature for paramagnetic ions, such as copper [38,39] (and also most lanthanide ions such as Eu^3+^, Dy^3+^, Ho^3+^, Er^3+^, Tm^3+^, and Yb^3+^) [40]; however, this is not the case for La^3+^, which is a diamagnetic ion [41]. Therefore, the broadening of the signals is not justified by the paramagnetic properties of the metal; in this case, the broadening may be the result of the lanthanum nucleus quadrupole moment. Nuclei that present nuclear spin (I) greater than 1/2, as is the case of ^139^La (I = 7/2), have a non-uniform distribution of charges due to their ellipsoidal shape. This asymmetry in the distribution of charges generates the quadrupole moment in ^139^La, which in turn contributes to a highly efficient relaxation mechanism and results in the broadening of the signals in the spectrum [42]. Although the CLA signals are wider in the spectrum of the complex, their integration was not affected by this effect, since the values of the integrals of the complex were similar when compared to the uncoordinated ligand.

The protons of the water molecule coordinated to the metal ion are hardly observed in the ^1^H NMR spectrum due to the high exchange rate between them [43,44], thus hindering the observation of its signal. This exchange rate, in addition to being affected by temperature, is influenced by the solvent used, since in the latter there may be protons available for exchange as well as a chemical environment that favors it. In this work, CDCl_3_ was used, and the ^1^H NMR experiment was performed at 25 °C. Under these conditions, the H_2_O signal in the form of traces was observed at 1.56 ppm [45]; however, in the spectrum of the complex, the H_2_O signal was observed at 3.09 ppm. Its attribution to coordination water was based on works published such as that by Diop and collaborators who reported the signal of water coordinated to tin in the complex [(2,6-CO_2_)_2_C_5_H_3_NSnBu_2_(H_2_O)_2_]CHCl_3_ at 3.43 ppm using the CDCl_3_ solvent [46]. Another work was reported by Minyaev and collaborators, who attributed the signal at 4.19 ppm to water coordinated to lanthanum also using the CDCl_3_ solvent [47].

The signals related to triethanolamine appear in the ^1^H NMR spectrum at 3.61 ppm (OCH_2_) and 2.67 ppm (NCH_2_) [48], that is, very close to the signals of uncoordinated CLA. The ^13^C NMR spectrum was acquired to confirm the absence of TEOA coordination to lanthanum in the inner coordination sphere of the lanthanum–CLA complex.

A comparison between the chemical shifts in the ^13^C NMR spectra obtained for CLA and the La(CLA)_2_(H_2_O)Cl complex is shown in Figure S2; the signal assignments are summarized in Table S3. The ^13^C NMR spectrum of the complex is remarkably similar to that of the free ligand, which is generally in good agreement with the ^1^H NMR data. The ^13^C NMR spectrum displays signals corresponding to the aromatic system (112.1, 129.6, 130.2, and 144.3 ppm), amino-substituent carbons (40.4 and 53.5 ppm), and the backbone chain (33.8, 26.4, and 33.6 ppm), along with a severely broadened carboxylate resonance at 180.3 ppm.

The first difference that can be noted between the spectra of the ligand and the complex is the change in the intensity of the signals related to carbons C-1, C-2, C-3 and C-4. The bond formed between CLA and lanthanum affects the relaxation time of the ^13^C and ^1^H nuclei, mainly those that are closest to the metal [49,50]. The presence of lanthanum affects the loss of magnetization via the spin-lattice relaxation process. The quadrupolar nucleus (La139) near C-1 impacts the T1 and T2 relaxation times of this carbon, thus contributing to the experimental spectral profile obtained for the La(CLA)_2_(H_2_O)Cl complex. The characteristics of the C-1 carbon signal, low intensity and broadened, constitute evidence of the formation of the coordination bond between La^3+^ and CLA via the carboxylic group. The low intensity of the signal related to the carbon closest to the coordination site with the metal is consistent with the ^13^C NMR spectrum of other complexes published [51,52].

The most relevant change is the chemical shift of carbon C-1 (from 179.9 ppm to 186.2 ppm). This change to a higher chemical shift value is consistent with the study published by Qin and collaborators, which reports the carbonyl shift to 181.16 ppm after complexation between CLA and platinum [13]. Thus, the ^13^C NMR spectra agree with the FTIR spectra and together confirm the coordination bond between La^3+^ and CLA via the carboxylic group of the ligand. The chemical shifts of carbons C-2, C-3 and C-4, which also showed a variation towards a higher chemical shift in the spectrum of the complex, prove the coordination between ligand and metal.

The signals of triethanolamine carbons in the ^13^C spectrum appear at 55.63 ppm (OCH_2_) and 58.85 ppm (NCH_2_) [48,53], which are absent in the ^13^C spectrum of La(CLA)_2_(H_2_O)Cl, which confirms the absence of this ligand in the synthesized complex.

The stoichiometry of the complex formed between CLA and La^3+^ was also proved by thermogravimetric analysis and elemental analysis.

The thermal analysis results from the collection of data on the thermal decomposition of the compounds studied; the records indicate the percentage of mass loss as a function of temperature.

**Figure 2:**
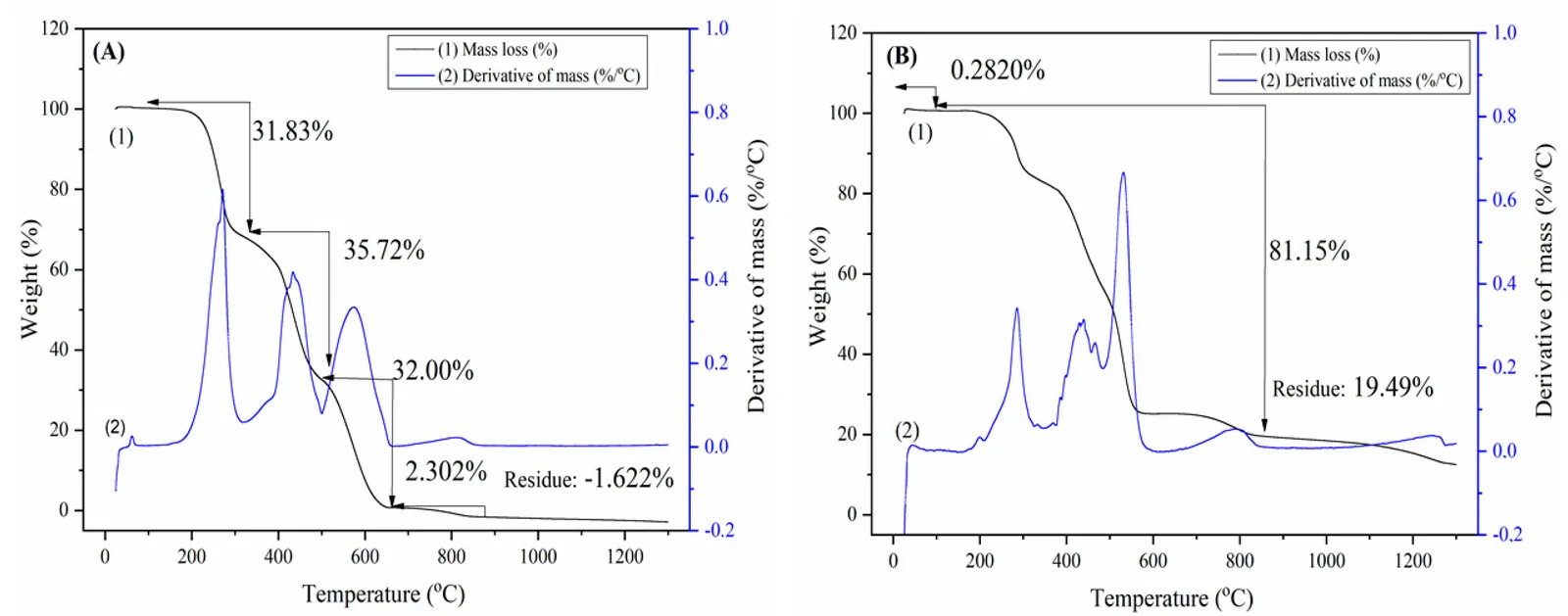
Thermogravimetric (TG) and derivative thermogravimetric (DTG) profiles of (**A**) chlorambucil (CLA) and (**B**) the lanthanum–chlorambucil complex [La(CLA)_2_(H_2_O)Cl].

The thermal analysis results for the CLA ligand and the La(CLA)_2_(H_2_O)Cl complex are shown in Figure 2.

The mass loss in the range 25–98 °C (Figure 2A) corresponds to hydrated water in the complex since the synthesis was carried out in an aqueous solution. The mass loss in the range between 98 and 209 °C corresponds to the coordinated water loss that occurs normally in the range of 150–200 °C [54]. The other mass loss observed between 200 and 860 °C occurs in similar temperature ranges for both the ligand and the complex.

The number of moles of lanthanum in the residue was calculated using an oxidizing atmosphere to get La_2_O_3_ after complete decomposition of the complex: n(La) = 6.97·10^−6^ moles. The same was done to determine the percentage of the ligand in the temperature range between 98 and 860 °C. A mass loss of 81.15% (4.73 mg) (Figure 2B) was recorded, which is equal to 1.55 × 10^−5^ mol of CLA (M = 304.212 g mol^−1^). Thus, the molar ratio between the ligand and metal is equal to 2.22; therefore, the thermal analysis of the complex formed between lanthanum and chlorambucil suggests a stoichiometry of 2:1 ligand:metal, which is in good agreement with CHN analysis data (Table 1).

Thus, using a set of different methods, we confirmed the formula of the synthesized lanthanum complex with CLA: La(CLA)_2_(H_2_O)Cl.

### 2.2. Theoretical Calculations

**Figure 3:**
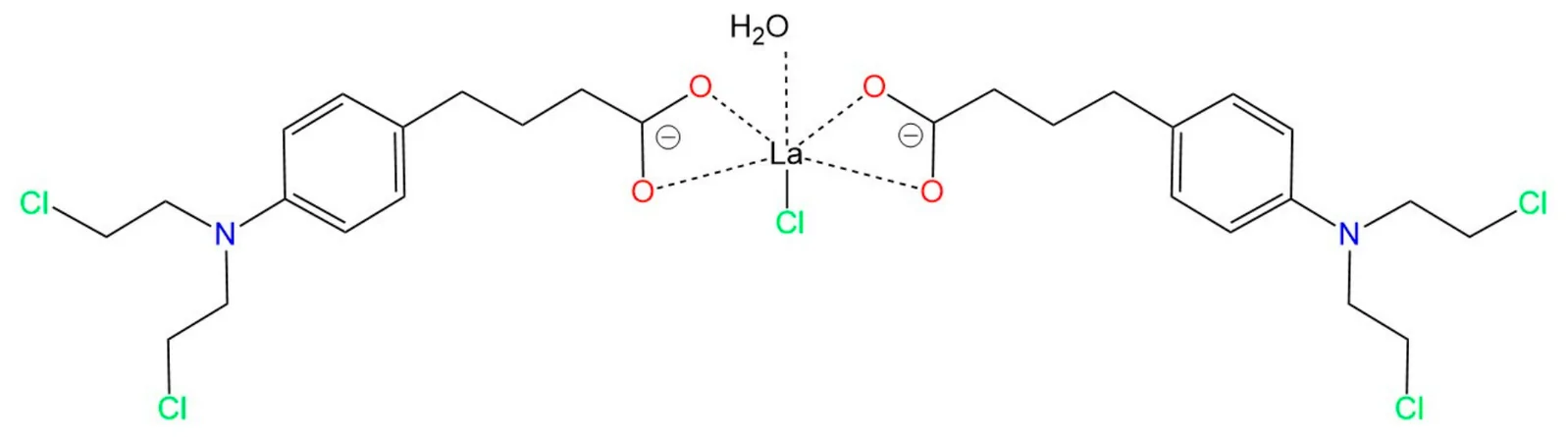
Proposed structure of La(CLA)_2_(H_2_O)Cl complex.

The synthesized lanthanum complex with chlorambucil, La(CLA)_2_(H_2_O)Cl, is amorphous, so it was not possible to grow single crystals suitable for X-ray structural analysis. The structure shown in Figure 3 is proposed based on spectral data. However, chlorambucil has wide coordination capabilities and thus can form different bonds with metal centers due to other donor atoms and functional groups. For example, the nitrogen atom or even chlorine atoms of chlorambucil are capable of coordinating to the metal center. In addition, the carboxyl group quite easily forms chelate complexes with lanthanides. To confirm the proposed structure, a quantum-chemical calculation was performed for the lanthanum complex with chlorambucil.

**Figure 4:**
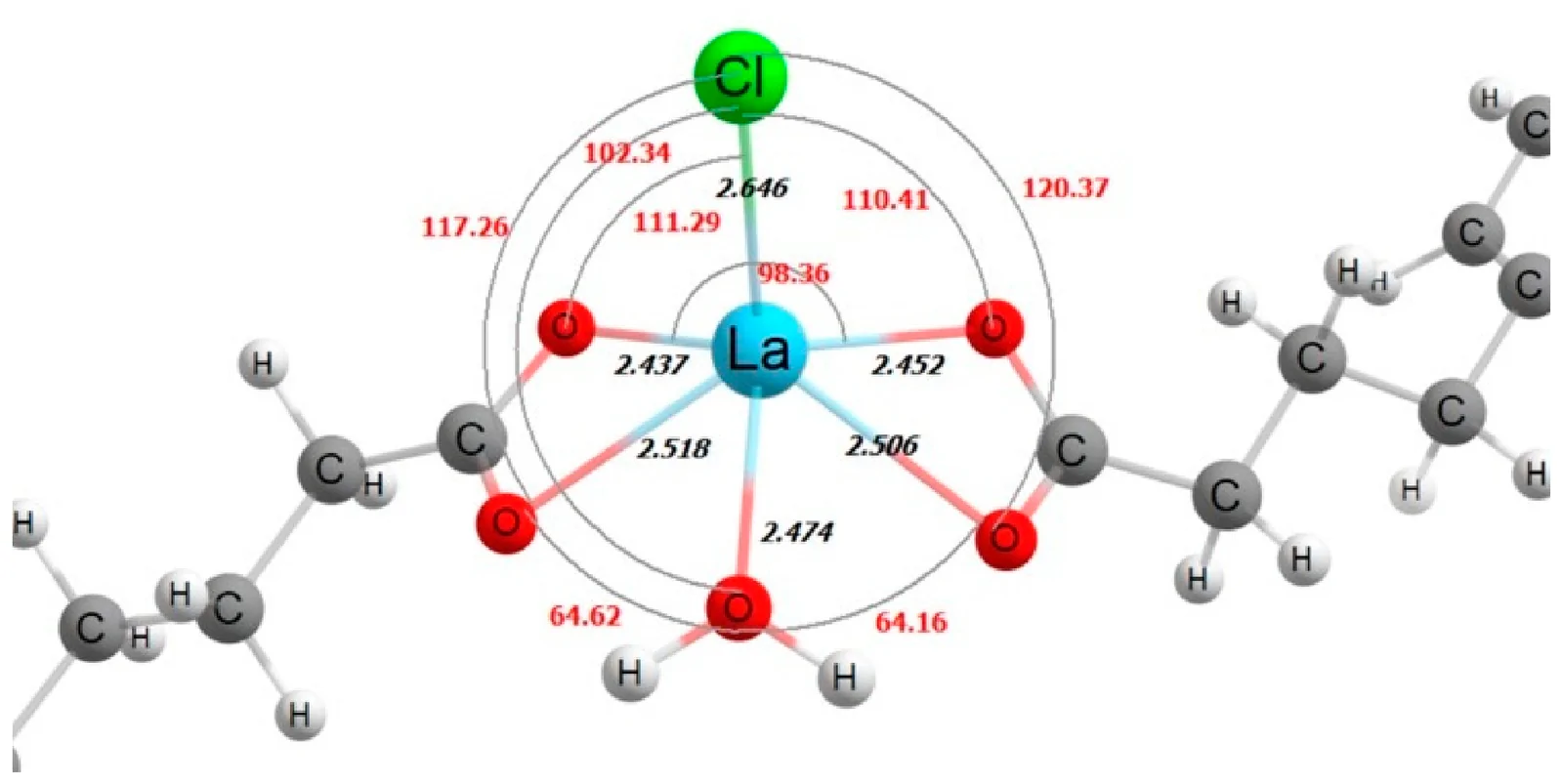
Fragment of calculated La(CLA)_2_(H_2_O)Cl structure with selected bond lengths and angles.

The functional Cam-B3LYP [55] and SDD basis set [56,57,58,59,60,61] were used for DFT calculation, which are quite good for describing the structures, spectral properties and other characteristics of complexes with different metal center natures [62,63,64]. The calculated structure of the La(CLA)_2_(H_2_O)Cl complex, the obtained coordinates of the atoms in the complex, and the calculated IR spectrum are given in the Supplementary Materials (Figures S3 and S4, Table S4). A fragment of the La(CLA)_2_(H_2_O)Cl structure with optimized geometry with selected angles and bond lengths is shown in Figure 4. The coordination number of the lanthanum atom is 6, which is quite typical for this metal with a geometry close to a distorted pentagonal pyramid (or strongly distorted octahedron geometry) with a chlorine atom in the vertex.

The bond lengths of the lanthanum with carboxyl group fall within the range of 2.437–2.518 Å, which is quite typical for such coordination [65]. The La-Cl bond length of 2.646 Å and the La-O_aq_ bond length of 2.474 Å are also quite typical for these ligands in the lanthanum coordination sphere [66,67]. Although the coordination number for lanthanum is equal to 6, a pentagonal pyramidal structure is not as common a geometry as an octahedron one; there are some examples when such a type of coordination occurs [68,69].

The calculated IR spectrum (Figure S4) is similar to the recorded one (Figure 1). The aggregate of these results and the absence of negative frequencies of normal vibrations allow us to assert that the calculated structure with optimized geometry corresponds to the synthesized complex.

### 2.3. Biological Tests

**Figure 5:**
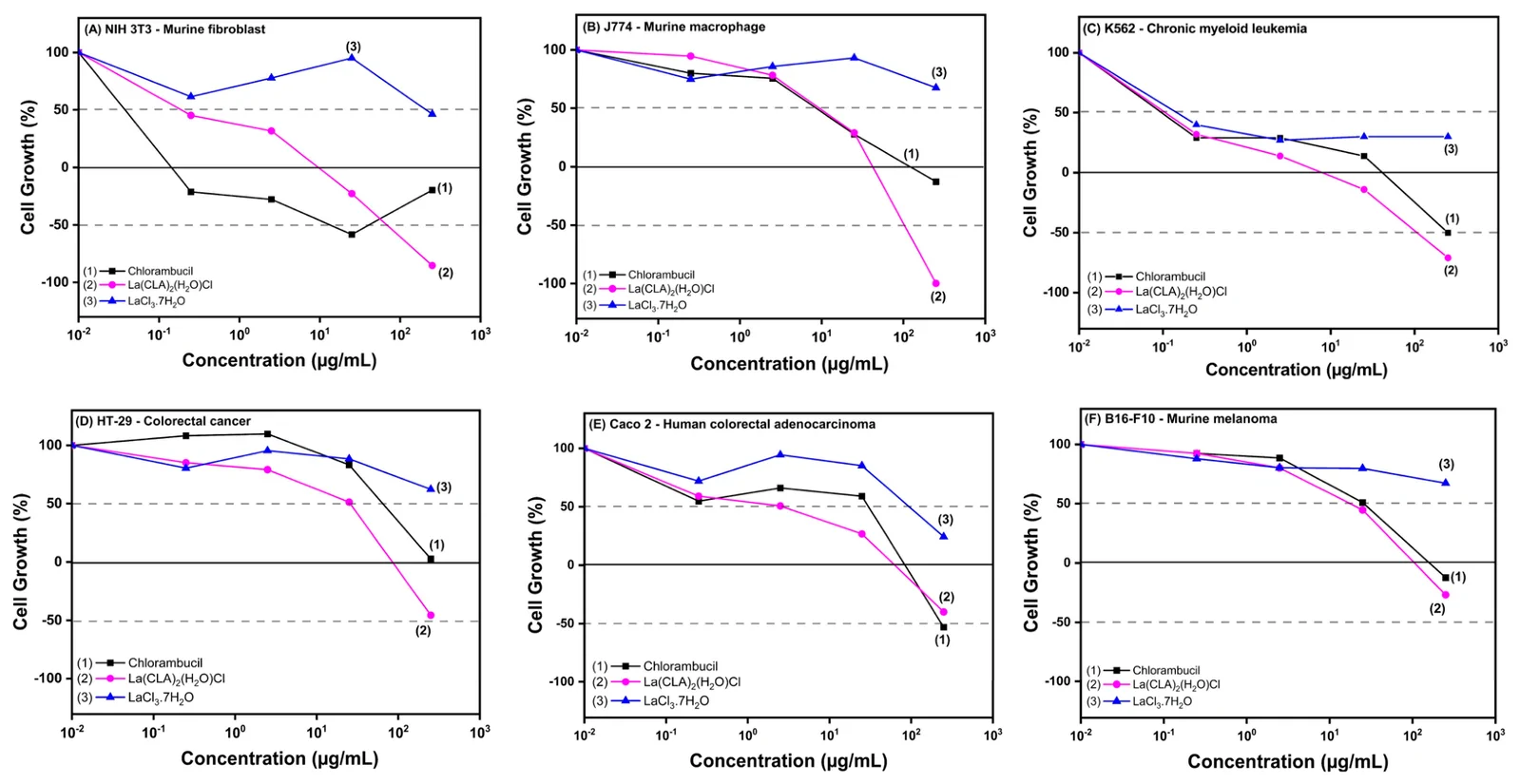
Cytotoxic activity evaluated by the MTT assay following 48 h exposure to chlorambucil (CLA), La(CLA)_2_(H_2_O)Cl, and LaCl_3_·7H_2_O from cell lines (**A**) NIH 3T3—murine fibroblast; (**B**) J774—murine macrophages; (**C**) K562—chronic myeloid leukemia; (**D**) HT-29—colorectal cancer; (**E**) Caco 2—human colorectal adenocarcinoma; and (**F**) B16-F10—murine melanoma.

Chlorambucil, La(CLA)_2_(H_2_O)Cl and LaCl_3_·7H_2_O were tested against the neoplastic cell lines K562 (chronic myeloid leukemia), HT-29 (colorectal cancer), Caco 2 (human colorectal adenocarcinoma) and B16-F10 (murine melanoma). For subsequent calculation of the selectivity index (SI) of the compounds, they were also tested against the normal cells NIH/3T3 (murine fibroblasts) and J774 (murine macrophages). The samples were tested at concentrations of 0.25, 2.5, 25 and 250 µg mL^−1^ and the cell viability results at each concentration can be seen in Figure 5.

The data can be evaluated from the perspective of inhibition of cell proliferation when the viability percentages are in the range between 0 and 100%, and the cytocidal effect is observed when the viability percentages are below zero.

Among the three samples tested, LaCl_3_·7H_2_O showed the lowest potential for inhibiting cell proliferation against all neoplastic cell lines. The La(CLA)_2_(H_2_O)Cl complex showed greater activity than CLA in the concentration range of 2.5–250 against the K562 and B16F10 cell lines. In the HT29 cell line, the complex showed greater activity than CLA in the entire concentration range tested, while against Caco 2, the results were better for La(CLA)_2_(H_2_O)Cl only at concentrations of 2.5 and 25 µg mL^−1^.

The values of TGI_50_ (100% cell growth-inhibitory concentration), GI_50_ (50% cell growth-inhibitory concentration) and LC_50_ (concentration that promotes 50% cell mortality) were calculated and the results are summarized in Table 2.

Chlorambucil (CLA), La(CLA)_2_(H_2_O)Cl and LaCl_3_·7H_2_O were evaluated against the neoplastic cell lines K562 (chronic myeloid leukemia), HT-29 (colorectal cancer), Caco-2 (human colorectal adenocarcinoma), and B16-F10 (murine melanoma). For the determination of the selectivity index (SI), the compounds were also tested in normal NIH/3T3 (murine fibroblasts) and J774 (murine macrophages) cells. The samples were evaluated at concentrations of 0.25, 2.5, 25, and 250 µg/mL, and the cell viability results are presented in Figure 5.

The results may be interpreted as indicative of cell proliferation inhibition when cell viability values range from 0 to 100%, whereas values below 0% are indicative of a cytocidal effect [70]. Among the evaluated samples, LaCl_3_·7H_2_O exhibited the lowest antiproliferative activity against all neoplastic cell lines tested, suggesting that the biological effects observed for the complexes are not exclusively associated with the lanthanum ion itself, but are likely related to the combined contribution of the metal center and the biologically active ligands.

Within the chlorambucil (CLA) series (Table 2), the La(CLA)_2_(H_2_O)Cl complex exhibited greater antiproliferative activity than the free ligand against the K562 and B16-F10 cell lines over the concentration range of 2.5–250 µg mL^−1^. For the HT-29 cell line, the superior activity of the complex was observed across the entire concentration range tested, whereas for Caco-2 cells this effect was detected only at the intermediate concentrations of 2.5 and 25 µg mL^−1^. These findings suggest that coordination of chlorambucil to the lanthanum ion enhances its antiproliferative activity, possibly as a result of changes in physicochemical properties such as stability, lipophilicity, and cellular uptake.

Similar findings have been reported in the literature for metal complexes containing chlorambucil. The most promising combination described to date was reported by Qin and co-workers [13], who synthesized two platinum complexes incorporating chlorambucil. The authors observed greater biological activity than that of the parent compounds in most of the cell lines evaluated. Notably, one of the complexes exhibited remarkable activity against cisplatin-resistant gastric carcinoma cells, with an IC_50_ value of 4.23 ± 0.47 µM, whereas chlorambucil and cisplatin displayed IC_50_ values of 81.83 ± 7.24 µM and 13.09 ± 1.21 µM, respectively. These results support the hypothesis that metal coordination can significantly enhance the antitumor activity of chlorambucil, in agreement with the behavior observed for the La(CLA)_2_(H_2_O)Cl complex in the present study.

The TGI, GI_50_, and LC_50_ values were calculated and are summarized in Table 2. Analysis of the GI_50_ values demonstrated that the pharmacological profile of chlorambucil was preserved following complexation with lanthanum, exhibiting greater activity against K562 chronic myeloid leukemia cells and lower activity against solid tumors. However, the lower GI_50_ values observed for the La(CLA)_2_(H_2_O)Cl complex compared with the free ligand indicate a consistent enhancement of antiproliferative activity following metal coordination.

In addition to its greater activity, the lanthanum complex exhibited higher selectivity toward the Caco-2 cell line and a selectivity index greater than 15 for the K562 cell line, a desirable characteristic in the development of novel antitumor agents. These findings suggest that coordination to lanthanum may enhance not only cytotoxic potency but also the ability to discriminate between tumor and normal cells.

The results obtained in the present study are supported by previous investigations involving lanthanum complexes with different bioactive ligands. Cârâc and co-workers reported a La^3+^ complex that exhibited activity against breast cancer (MCF-7) and ovarian cancer (A2780) cells, with IC_50_ values ranging from 1.4 to 70.0 µM [23]. Similarly, Yang and co-workers synthesized lanthanum complexes that displayed greater activity than their organic precursors against cisplatin-resistant lung adenocarcinoma (A549) cells, with IC_50_ values ranging from 0.025 to 0.097 µM, while also exhibiting low toxicity toward normal HL-7702 cells [24]. Collectively, these studies corroborate the findings of the present work and highlight the potential of lanthanum complexes as a promising strategy for the development of novel antineoplastic agents capable of exhibiting enhanced biological activity and, in some cases, improved selectivity compared with the corresponding free drugs.

## 3. Materials and Methods

Commercial-grade chlorambucil (Sigma-Aldrich, Burlington, MA, USA), triethanolamine (Synth, Diadema, Brazil, 99.5%), LaCl_3_·7H_2_O (Sigma-Aldrich 70%), ethanol (EMSURE, Darmstadt, Germany, 99.9%), potassium hydroxide (Dinamica, Indaiatuba, Brazil, 85%), hydrochloric acid (Sigma-Aldrich, 37%), chloroform (Sigma-Aldrich, 99.5%), sodium hydroxide (Proquimios, Bogotá, Colombia, 97%), dimethyl sulfoxide (Sigma-Aldrich, ≥99.9%), DMSO-d^6^ (Sigma-Aldrich, 99.9%) and CDCl_3_ (Sigma-Aldrich, ≥99%) were used without additional purification.

IR spectra were recorded using a Thermo Fischer Scientific Nicolet iS5 infrared spectrophotometer. For analysis, KBr was previously dried in an oven at 120 °C for 12 h. The material was macerated together with KBr and the mixture was pressed to obtain a tablet, which was used in the spectrophotometer. The TGA measurement was performed on TGA SDTA 851 thermogravimetric analysis equipment (METTLER TOLEDO, Greifensee, Switzerland). For the analyses, a temperature range between 25 and 1300 °C was used with a heating rate of 10 °C/minute, in a synthetic air atmosphere with a flow of 70 mL/min. The data obtained were processed using OriginPro 9.0 software. Elemental analysis was performed using the Elemental Analyzer equipment—Perkin Elmer 2400 series II at the Analytical Center—USP. The MNR spectra were recorded on Bruker DPX-300 (300/75 MHz) and Bruker Avance Neo 500 (500/125 MHz) nuclear magnetic resonance spectrometers; the data obtained were processed in the MestReNova 12.0.0-20080 software.

### 3.1. Synthesis of the La(CLA)_2_(H_2_O)Cl Complex

**Figure 6:**
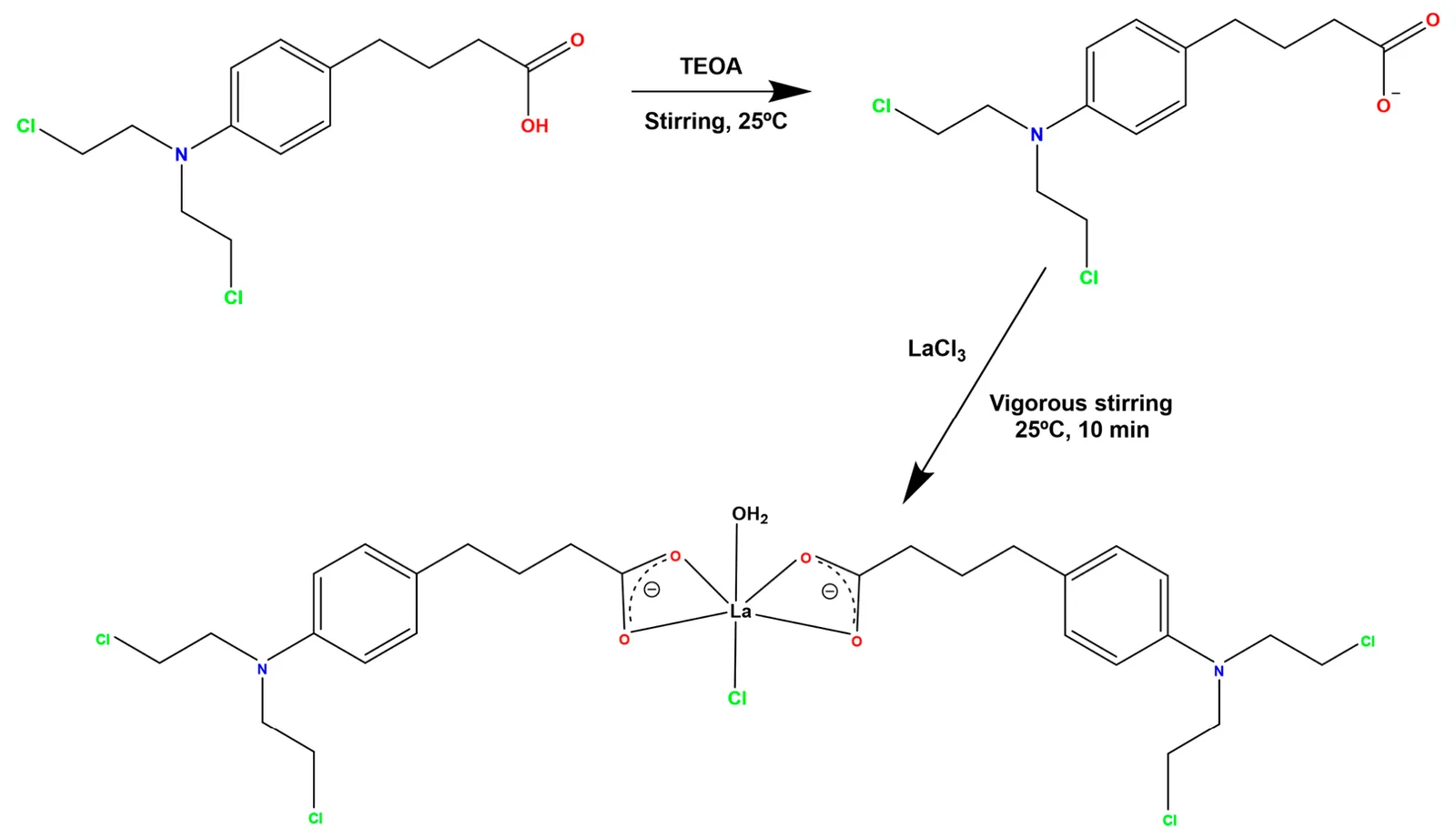
Proposed synthetic route for the preparation of the lanthanum–chlorambucil (La–CLA) coordination complex through deprotonation of chlorambucil with triethanolamine (TEOA), followed by coordination with lanthanum chloride under stirring and heating conditions.

In a beaker, 61 mg (0.20 mmol) of CLA was suspended in 1 mL of deionized water and kept under vigorous stirring, and then 400 µL of an aqueous solution of 1mM triethanolamine was added over the ligand until its complete dissolution at pH ≅ 6. In another beaker, 36 mg of LaCl_3_·7H_2_O (0.06 mmol) was dissolved in 1.5 mL of deionized water and then added dropwise to the CLA solution. The white solid formed immediately. The reaction was kept under vigorous stirring for 10 min at room temperature. The solid obtained was centrifuged and washed with deionized water, and at the end of the washings, the product was lyophilized. This drying method was used to avoid hydrolysis of the chlorine atoms present in the complex [71]. The yield of this reaction was 92.4% and the synthesis scheme is shown in Figure 6.

### 3.2. Theoretical Calculations

The calculations were performed using the Gaussian 16 program (revision A.03) [72] using computational resources provided by Superintendência de Tecnologia da Informação da Universidade de São Paulo. All structures were fully optimized; the energy minima were checked by analytical calculation of the frequencies of normal vibrations. Geometry optimizations and calculations of the frequencies of normal vibrations were performed at the DFT level using the Cam-B3LYP functional [55]. The SDD basis set was chosen for the lanthanum atom [56,57,58,59,60,61]. For the remaining elements, the 6-31G basis set was used [73,74,75,76]. The Chemcraft program was used to visualize the optimized geometries and normal vibrations [77].

### 3.3. In Vitro Anticancer Assay

The cell lines K562 (ATCC^®^ CCL-243™, chronic myeloid leukemia), HT-29 (ATCC^®^ HTB-38™, colorectal cancer), Caco 2 (human colorectal adenocarcinoma) and B16-F10 (ATCC^®^ CRL-6475™, murine melanoma) were used to perform the tests. The normal cell lines NIH/3T3 (ATCC–CRL 1658, murine fibroblast) and J774 (ATCC^®^ TIB-67™, murine macrophages) were also used. The cells were cultured in Dulbecco’s Modified Eagle’s Medium (DMEM) supplemented with 10% fetal bovine serum (FBS) and 1% antibiotic (penicillin/streptomycin). The cells were kept in an incubator at 37 °C with 5% CO_2_.

The synthesized complexes were diluted in DMSO (100 µg mL^−1^). To add the materials to the cells, a serial dilution was made using culture medium; the concentrations used were 0.25, 2.5, 25 and 250 µg mL^−1^.

After the addition of the test samples, the cells were kept exposed for 48 h in an incubator. After the incubation period, the culture medium containing the treatment was discarded, followed by subsequent addition of MTT solution in DMEM (10% MTT in PBS 5mg mL^−1^, and 90% DMEM) and incubation in an oven for 4 h. The next step consisted of aspirating the supernatant medium from the wells and adding DMSO to dilute the formazan crystals. Subsequently, the absorbance spectra were recorded at a wavelength of 540 nm, to quantify cell viability in a Molecular Devices SpectraMax 190^®^ microplate reader.

The MTT colorimetric assay is a standard test that measures cell viability commonly used to verify the cytotoxicity of substances. MTT is a yellow salt (3-(4,5-dimethylethylthiazol-2-yl)-2,5-diphenyltetrazolium bromide). Viable cells with active metabolism convert MTT into purple-colored product formazan. Nonviable cells lose the ability to convert MTT into formazan [78]; therefore, the formazan concentration at the end of the test is directly proportional to the number of viable cells. The data obtained were processed using Origin Version 6.0 software to determine the 50% inhibitory concentration of cell growth (GI_50_), which was calculated by nonlinear regression analysis. The selectivity index (SI) of the test samples was determined using the ratio between the GI_50_ values obtained against normal cell lines (NIH/3T3 and J774) and the GI_50_ value obtained against neoplastic cells, as exemplified in the following equation:

$$\mathrm{IS}=\frac{{\mathrm{GI}}_{50}\;\mathrm{NIH}/3T3}{{\mathrm{GI}}_{50}\;\mathrm{neoplastic}\;\mathrm{cells}}\;\mathrm{and IS}=\frac{{\mathrm{GI}}_{50}\;J774}{{\mathrm{GI}}_{50}\;\mathrm{neoplastic}\;\mathrm{cells}\;}$$

## 4. Conclusions

Results obtained using NMR and FTIR techniques for the complex formed between La^3+^ and the CLA ligand suggest that coordination occurred through the –COO^−^ group; the data further indicate that the TEOA base used during synthesis is not part of the formed complex’s coordination sphere. Thermogravimetric analysis indicated a ligand-to-metal molar ratio of 2:1 for the produced material; by cross-referencing TG data with information inferred from C, H, N, and Cl elemental analysis, structural possibilities were narrowed down, identifying the structure La(CLA)_2_(H_2_O)Cl as the most consistent with the experimental data. Cell viability assay results showed that the complex exhibited a lower GI_50_ value than the ligand across all tested neoplastic cell lines, indicating higher activity than its precursor, CLA. Selectivity index (SI) values for both the complex and CLA were below 1.54 for the B16-F10, HT-29, and Caco-2 cell lines; however, SI results for the K562 cell line were 15.64 for the complex and 67.02 for CLA.

## Figures and Tables

**Table 1:** Elemental analysis of carbon, hydrogen, nitrogen and chorine of La(CLA)_2_(H_2_O)Cl complex.

	%C	%H	%N	%Cl
Measure 1	45.83	4.45	3.93	22.03
Measure 2	46.03	4.66	3.79	22.15
Measure 3	45.93	4.56	3.86	22.09
Calculated	42.10	4.80	3.51	22.19

**Table 2:** TGI_50_, GI_50_ and LC_50_ for chlorambucil (CLA), La(CLA)_2_(H_2_O)Cl, LaCl_3_.7H_2_O and doxorubicin in K562, B16F10, HT29, CACO-2, J774 and NIH/3T3 cell lines during 48 h exposure.

Sample	TGI50 μM (μg/mL)	GI50 μM (μg/mL)	LC50 μM (μg/mL)	Selectivity Index (SI)	
				J774	NIH-3T3
*K562 (ATCC CCL-243)—Chronic Myeloid Leukemia*					
Chlorambucil	103.24 (31.41)	0.80 (0.24)	195.74 (59.55)	67.02	0.33
La(CLA)_2_(H_2_O)Cl	3.70 (2.96)	0.31 (0.24)	8.53 (6.81)	15.64	1.01
LaCl_3_.7H_2_O	9.51 (3.53)	0.66 (0.25)	>673.18 (>250)	1583.29	939.77
Doxorubicin	0.59 (0.32)	0.05 (0.03)	0.89 (0.48)	0.39	9.19
*B16-F10 (ATCC CRL6475)—Murine melanoma*					
Chlorambucil	121.23 (36.88)	83.26 (25.33)	272.00 (82.74)	0.64	0.00
La(CLA)_2_(H_2_O)Cl	45.62 (36.44)	28.66 (22.89)	>312.98 (>250)	0.17	0.01
LaCl_3_·7H_2_O	1668.90 (619.78)	1046.83 (388.76)	>673.18 (>250)	1.01	0.60
Doxorubicin	0.47 (0.25)	0.05 (0.03)	1.11 (0.60)	0.40	9.33
*HT-29 (ATCC HTB38)—Colorectal cancer*					
Chlorambucil	832.68 (253.31)	103.95 (31.62)	>821.79 (>250)	0.52	0.00
La(CLA)_2_(H_2_O)Cl	52.19 (41.69)	31.86 (25.45)	74.18 (59.25)	0.15	0.01
LaCl_3_·7H_2_O	1269.02 (471.27)	852.88 (316.74)	>673.18 (>250)	1.23	0.73
Doxorubicin	0.50 (0.27)	0.05 (0.03)	>459.96 (>250)	0.40	9.32
*CACO-2—Human colorectal adenocarcinoma*					
Chlorambucil	496.15 (150.93)	108.45 (32.99)	818.77 (249.08)	0.49	-
La(CLA)_2_(H_2_O)Cl	44.61 (35.63)	3.11 (2.48)	>312.98 (>250)	1.54	0.10
LaCl_3_·7H_2_O	815.91 (303.01)	432.22 (160.51)	>673.18 (>250)	2.44	1.45
Doxorubicin	0.73 (0.40)	0.39 (0.21)	4.66 (2.54)	0.05	1.12
*J774 (ATCC TIB-67)—Murine macrophages*					
Chlorambucil	105.06 (31,96)	53.57 (16.30)	118.26 (35.97)	-	-
La(CLA)_2_(H_2_O)Cl	38.70 (30,91)	4.78 (3.82)	83.39 (66.61)	-	-
LaCl_3_·7H_2_O	1620.41 (601.77)	1052.84 (390.99)	-	-	-
Doxorubicin	0.02 (0.01)	0.02 (0.01)	0.02 (0.01)	-	-
*NIH/3T3 (ATCC CRL-1658TM)*					
Chlorambucil	0.80 (0.24)	0.27 (0.08)	8.39 (2.55)	-	-
La(CLA)_2_(H_2_O)Cl	4.69 (3.74)	0.31 (0.25)	7.92 (6.33)	-	-
LaCl_3_·7H_2_O	1136.50 (422.06)	624.92 (232.08)	>673.18 (>250)	-	-
Doxorubicin	3.99 (2.17)	0.43 (0.24)	4.80 (2.61)	-	-

## Data Availability

The original contributions presented in this study are included in the article/Supplementary Materials. Further inquiries can be directed to the corresponding author. Acknowledgments: The authors acknowledge the Universidade Federal de Mato Grosso do Sul, Brasil (UFMS); Coordenação de Aperfeiçoamento de Pessoal de Nível Superior, Brasil (CAPES); Conselho Nacional de Desenvolvimento Científico e Tecnológico, Brasil (CNPq); and Fundação de Apoio ao Desenvolvimento do Ensino, Ciência e Tecnologia do Estado de Mato Grosso do Sul, Brasil (FUNDECT-MS) for their support. Sabrina Epifânio Soares de Souza, Brunna Mercedes Barboza Borges, Guilherme Pereira Biazoto, Diego Boldo and Vasilii Khripun thank the CAPES, CNPq and FUNDECT for the scholarships.

## Supplementary Materials

The following supporting information can be downloaded at https://www.mdpi.com/article/10.3390/molecules31203584/s1: Figure S1: Comparison between the ^1^H NMR spectra of chlorambucil (CLA) (top) and the lanthanum–chlorambucil complex [La(CLA)_2_(H_2_O)Cl] (bottom), recorded in CDCl_3_; Figure S2: ^13^C NMR spectra of (A) chlorambucil (CLA) and (B) the lanthanum–chlorambucil complex [La(CLA)_2_(H_2_O)Cl] recorded in CDCl_3_, showing the chemical shift assignments of the carbon atoms and the coordination-induced displacement of the carboxylate carbon signal; Figure S3: Calculated structure for [La(CLA)_2_(H_2_O) Cl] complex; Figure S4: Calculated (up) and experimental (bottom) IR spectra for [La(CLA)_2_(H_2_O)Cl] complex; Table S1: Assignment of the bands observed in the infrared spectra of the triethanolamine, the La(CLA)_2_(H_2_O)Cl complex (experimental and calculated), and chlorambucil, and comparison with literature data for the ligand; Table S2: Comparison of the ^1^H NMR chemical shifts (δ, ppm) of chlorambucil (CLA) and the lanthanum–chlorambucil complex [La(CLA)_2_(H_2_O)Cl], experimental multiplicities, coupling constants (J), integrals, and the corresponding chemical shift variations (Δ ppm); Table S3: ^13^C NMR data for chlorambucil (CLA) and complex La(CLA)_2_(H_2_O)Cl; Table S4: Coordinates of the atoms for calculated [La(CLA)_2_(H_2_O)Cl] complex.

## Abbreviations

The following abbreviations are used in this manuscript:

DMSO-d^6^	Deuterated dimethyl sulfoxide
GI_50_	Concentration required to inhibit 50% of cell growth
CLA	Chlorambucil
SI	Selectivity index
CDCl_3_	Deuterated chloroform
