# Supplementary material for: Synthesis, Characterization and In Vitro Cytotoxic Assay of La(III) Complex with Chlorambucil
Source: Molecules. 2026 Oct 9;31(20):3584. doi: 10.3390/molecules31203584 (PMC13707427; doi:10.3390/molecules31203584)
Supplement: Supplementary file 1 [file molecules-31-03584-s001.zip › molecules-4588197-supplementary.pdf]

Supplementary Information

Synthesis, Characterization and *In Vitro* Cytotoxic Assay of La(III) Complex with Chlorambucil

Fernanda Timoteo, Kristiane Fanti Del Pino, Danielle Bogo, Diego Boldo, Vasilii Khripun, Franciele Garcia Baveloni, Sabrina Epifânio Soares de Souza, Guilherme Pereira Biazoto, Brunna Mercedes Barboza Borges, Adriana Pereira Duarte, Adriana da Silva Posso and Marco Antonio Utrera Martines

Table S1. Assignment of the bands observed in the infrared spectra of the triethanolamine, the La(CLA)$_2$(H$_2$O)Cl complex (experimental and calculated), and chlorambucil, and comparison with literature data for the ligand.

Attribution	Absorbtion (cm^{-1})				
	Clorambucil (Experimenal)	Clorambucil [28-30]	Trietanolamina	Complex La(CLA)$_2$(H$_2$O)Cl (experimental)	Complex Calc.
**ν O-H**	3442	3360	3382	3423	3807, 3660
**ν C-H (sp^2)**	3080	3079	-	3095	3210-3267
**ν C-H (sp^3)**	2931	2932	2952	2933	3053-3180
**δ C=O**	1705	1697	-		
**ν_{ass} C=C (sp^2)**	1612	1613	-	1612	1603
**ρ_{ass} H-C-H**	1518	1517	1458	1520	1522-1538
**ν_s COO$^-$<br>ν_{as} COO$^-$<br>$\Delta\nu = \nu_{as}-\nu_s$**				1470<br>1610<br>140	1426<br>1580<br>154
**ν_s C=C (sp^2)**	1456	1456	-	1439	1395
**ν_s C-N**	1252	1251	1033	1250	1262, 1269
**ν_s C-Cl**	561	552	-	561	694
**ν La-O**				525	510, 529

Figure S1. Comparison between the ¹H NMR spectra of chlorambucil (CLA) (top) and the lanthanum–chlorambucil complex $[La(CLA)_2(H_2O)Cl]$ (bottom), recorded in $CDCl_3$.

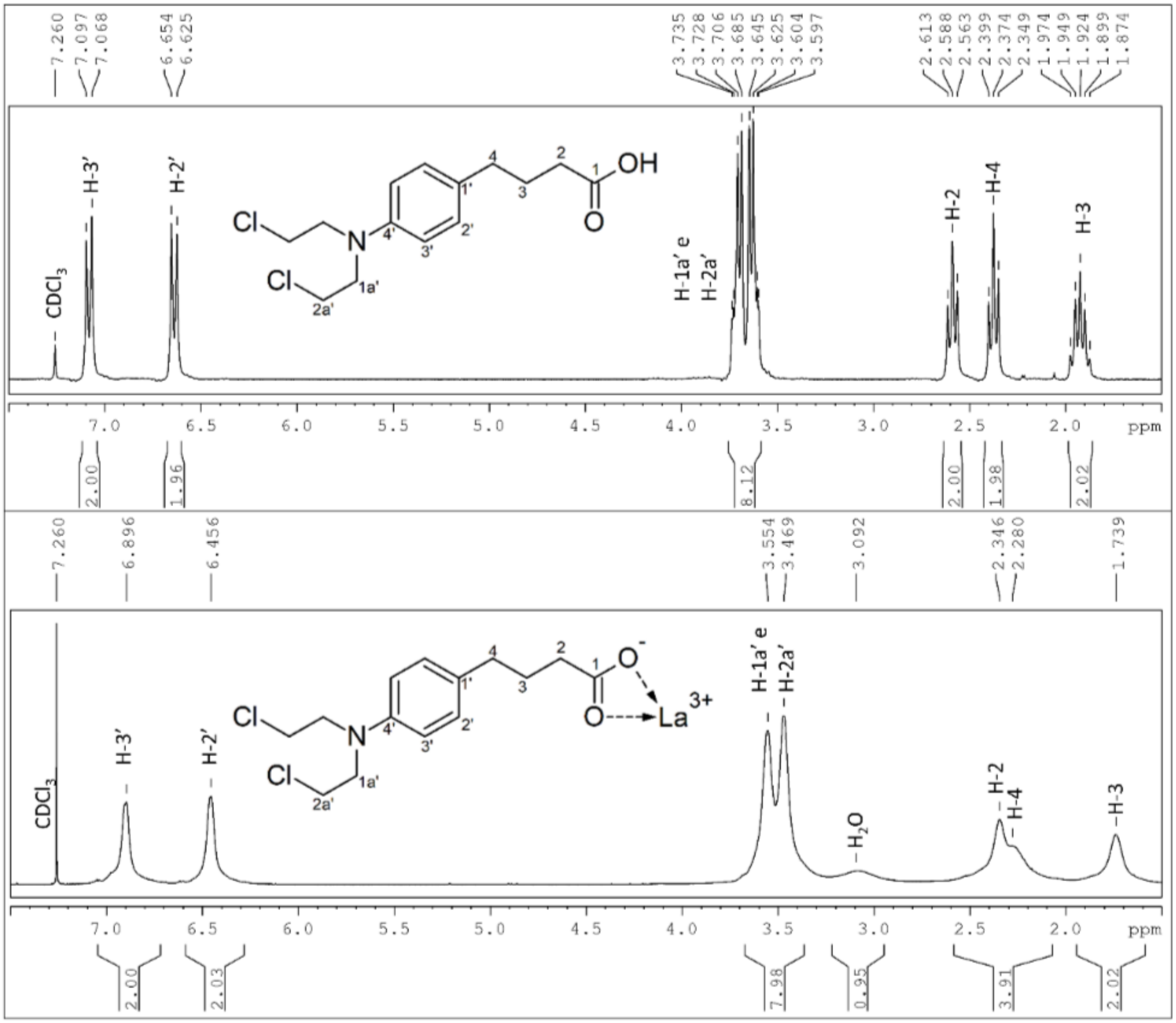

Table S2. Comparison of the ^{1}H NMR chemical shifts (δ, ppm) of chlorambucil (CLA) and the lanthanum–chlorambucil complex [La(CLA)$_2$(H$_2$O)$_2$Cl], experimental multiplicities, coupling constants (J), integrals, and the corresponding chemical shift variations (Δ ppm).

Chemical shift (ppm)				
Hydrogen	Chlorambucil [30,68] (Mohamad)	Chlorambucil Experimental (multiplicity, integrals)	La(CLA)$_2$(H$_2$O)$_2$Cl Experimental (integrals)	Δ ppm[1]
H-2	1.8-2.8	2.59 (t., J=7,5 Hz, 2H)	2.34 (2H)	-0.25
H-3	1.8-2.8	1.92 (quint., J=7,5 Hz, 2H)	1.74 (2H)	-0.18
H-4	1.8-2.8	2.37 (t., J=7,5 Hz, 2H)	2.28 (2H)	-0.09
H-2'	6.6-7.1	6.62 (d., J=8,6 Hz, 2H)	6.45 (2H)	-0.17
H-3'	6.6-7.1	7.07 (d., J=8,6 Hz, 2H)	6.89 (2H)	-0.18
H-1a'	3.6	3.59-3.73 (m, 8H)	3.47-3.55 (8H)	-0.12 - 0.18
H-2a'	3.6	3.59-3.73 (m, 8H)	3.47-3.55 (8H)	-0.12 - 0.18
H_2O coordinated	-	-	3.09 (1H)	-

[1]Difference between chemical shift of complex and ligand hydrogen signals.

Figure S2. ^{13}C NMR spectra of **(A)** chlorambucil (CLA) and **(B)** the lanthanum–chlorambucil complex [La(CLA)$_2$(H_2O)$_2$Cl] recorded in $CDCl_3$, showing the chemical shift assignments of the carbon atoms and the coordination-induced displacement of the carboxylate carbon signal.

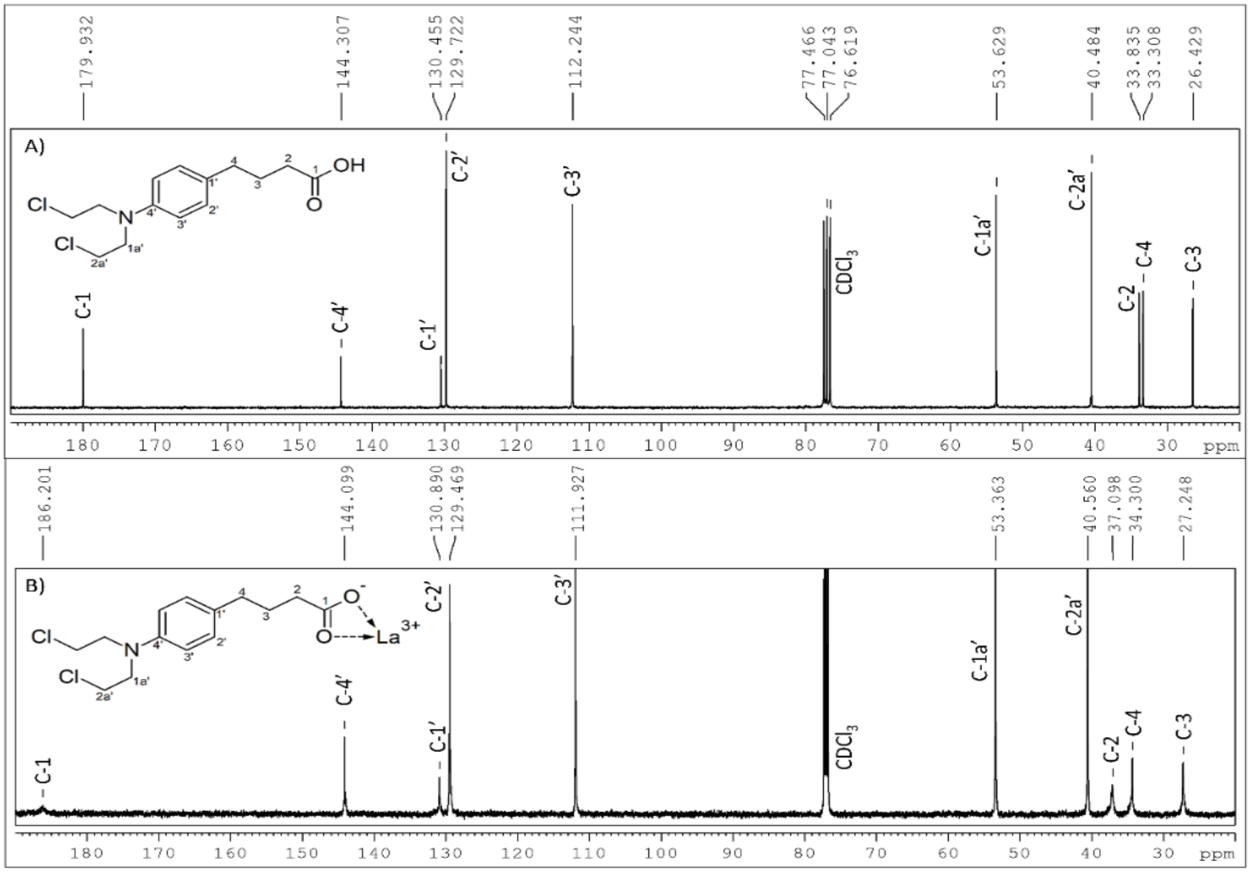

Tabel S3. ^{13}C NMR data for Chlorambucil (CLA) and complex $La(CLA)_2(H_2O)Cl$.

Chemical shift (ppm)				
Carbon	Clorambucil [31]	Clorambucil (Experimental)	$La(CLA)_2(H_2O)Cl$ (Experimental)	Δ ppm
C-1	180.3	179.9	186.2	6.3
C-2	33.8	33.8	37.1	3.3
C-3	26.4	26.4	27.2	0.8
C-4	33.4	33.3	34.3	1
C-1’	130.2	130.5	130.9	0.4
C-2’	129.6	129.7	129.4	-0.3
C-3’	112.1	112.2	111.9	-0.3
C-4’	144.3	144.3	144.1	-0.2
C-1a’	53.5	53.6	53.3	-0.3
C-2a’	40.4	40.5	40.6	0.1

Figure S3. Calculated structure for $[La(CLA)_2(H_2O)Cl]$ complex

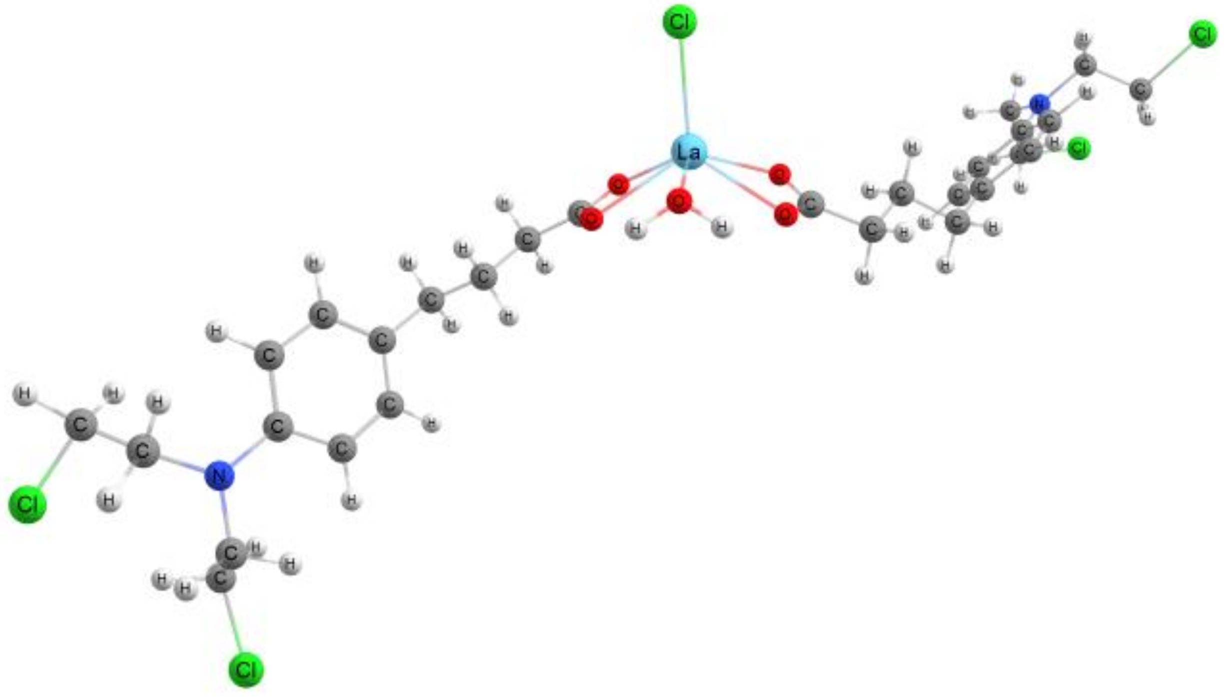

Figure S4. Calculated (up) and experimental (bottom) IR spectra for $[La(CLA)_2(H_2O)Cl]$ complex

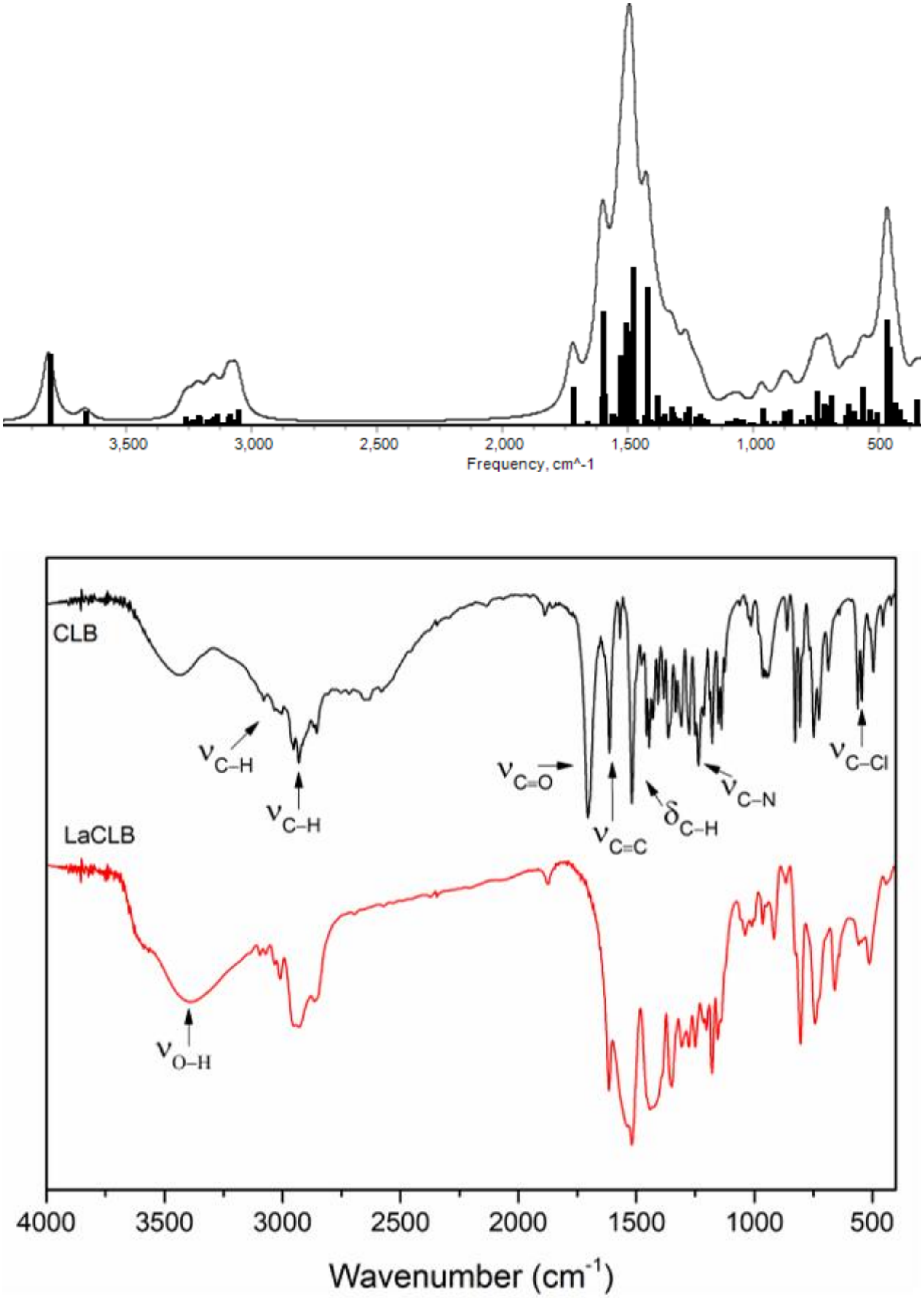

Table S4. Coordinates of the atoms for calculated [La(CLA)$_2$(H$_2$O)Cl] complex.

Cl	0.597038000	-4.544383000	0.505848000
O	-1.212827000	-2.134897000	-2.109274000
C	-7.116041000	0.358458000	1.542107000
C	-7.992429000	-0.727438000	1.512388000
C	-7.493564000	1.499807000	0.831199000
C	-9.189925000	-0.685454000	0.806428000
C	-8.685066000	1.561437000	0.118431000
C	-9.564308000	0.462010000	0.081500000
N	-10.760794000	0.508804000	-0.652530000
C	-11.104507000	1.698403000	-1.425271000
C	-11.584335000	-0.681764000	-0.786523000
C	-12.500228000	-0.984428000	0.390638000
Cl	-13.972330000	0.175575000	0.440223000
C	-11.691631000	2.800940000	-0.551350000
Cl	-11.932427000	4.342631000	-1.588028000
C	-5.800713000	0.288994000	2.280150000
C	-4.655918000	-0.251084000	1.405504000
C	-3.334341000	-0.315524000	2.166491000
C	-2.185537000	-0.853440000	1.371377000
O	-2.331106000	-1.265860000	0.152659000
O	-1.003826000	-0.924136000	1.902701000
C	6.957735000	1.081229000	-1.188514000
C	7.223424000	1.792550000	-0.016919000
C	7.974142000	0.258339000	-1.677245000
C	8.442723000	1.690056000	0.643055000
C	9.199798000	0.141503000	-1.031880000

C	9.463240000	0.851348000	0.154847000
N	10.690792000	0.722994000	0.825377000
C	11.721752000	-0.172198000	0.309060000
C	10.881274000	1.340963000	2.127298000
C	11.238781000	2.820074000	2.107632000
Cl	13.016319000	3.097275000	1.578398000
C	12.498089000	0.454765000	-0.843320000
Cl	13.676744000	-0.815885000	-1.554411000
C	5.615963000	1.172677000	-1.874911000
C	4.556824000	0.259574000	-1.234307000
C	3.209330000	0.351441000	-1.952437000
C	2.118863000	-0.435705000	-1.295105000
O	2.144397000	-0.689597000	-0.025986000
O	1.103555000	-0.864018000	-1.981247000
La	0.072796000	-2.000611000	0.000214000
H	-2.141039000	-1.860736000	-1.996271000
H	-0.742306000	-1.680470000	-2.832419000
H	-7.741125000	-1.630625000	2.060551000
H	-6.844097000	2.370129000	0.836618000
H	-9.828419000	-1.557723000	0.834283000
H	-8.920689000	2.476329000	-0.407525000
H	-10.224762000	2.075579000	-1.957468000
H	-11.836505000	1.419690000	-2.185829000
H	-12.187601000	-0.587781000	-1.692463000
H	-10.940593000	-1.559772000	-0.933836000
H	-12.931709000	-1.981275000	0.313812000
H	-12.002961000	-0.856460000	1.349028000

H	-12.671092000	2.536554000	-0.164229000
H	-11.027735000	3.088362000	0.260398000
H	-5.530824000	1.286688000	2.649294000
H	-5.906510000	-0.350497000	3.165839000
H	-4.916223000	-1.247754000	1.033334000
H	-4.542877000	0.381897000	0.518321000
H	-3.039112000	0.674548000	2.536780000
H	-3.426602000	-0.942765000	3.063028000
H	6.462416000	2.450541000	0.392250000
H	7.809612000	-0.307033000	-2.589523000
H	8.587273000	2.275776000	1.540496000
H	9.945469000	-0.512041000	-1.463126000
H	12.415188000	-0.407427000	1.118738000
H	11.279360000	-1.120861000	-0.013118000
H	9.964045000	1.234317000	2.722418000
H	11.660150000	0.793129000	2.662851000
H	10.638443000	3.384170000	1.398038000
H	11.170819000	3.260031000	3.101433000
H	11.853469000	0.755965000	-1.665557000
H	13.110303000	1.289607000	-0.515549000
H	5.254387000	2.209336000	-1.848737000
H	5.727087000	0.910097000	-2.934450000
H	4.914237000	-0.776585000	-1.247487000
H	4.423477000	0.522222000	-0.180443000
H	2.875537000	1.399226000	-1.985123000
H	3.279406000	0.022081000	-2.993750000
